# Energy-Efficient Trajectory Optimization for UAV-Based Hybrid FSO/RF Communications with Buffer Constraints

**DOI:** 10.3390/e23121596

**Published:** 2021-11-28

**Authors:** Rong-Rong Lu, Yang Ma, Sheng-Hong Lin, Bingyuan Zhang, Qinglin Wang, Jin-Yuan Wang

**Affiliations:** 1 Key Laboratory of Broadband Wireless Communication and Sensor Network Technology, Nanjing University of Posts and Telecommunications, Nanjing 210003, China; 1019010205@njupt.edu.cn (R.-R.L.); 2018010201@njupt.edu.cn (S.-H.L.); 2 National Mobile Communications Research Laboratory, Southeast University, Nanjing 210096, China; yangma@seu.edu.cn; 3 Department of Automation Engineering, Nanjing Institute of Mechatronic Technology, Nanjing 211306, China; 4 Shandong Key Laboratory of Optical Communication Science and Technology, Liaocheng University, Liaocheng 252059, China; byzhang@lcu.edu.cn (B.Z.); wangqinglin@lcu.edu.cn (Q.W.)

**Keywords:** energy efficiency, FSO/RF communications, trajectory optimization, UAV

## Abstract

This paper focuses on an unmanned aerial vehicle (UAV) assisted hybrid free-space optical (FSO)/radio frequency (RF) communication system. Considering the rate imbalance between the FSO and RF links, a buffer is employed at the UAV. Initially, theoretical models of energy consumption and throughput are obtained for the hybrid system. Based on these models, the theoretical expression of the energy efficiency is derived. Then, a nonconvex trajectory optimization problem is formulated by maximizing the energy efficiency of the hybrid system under the buffer constraint, velocity constraint, acceleration constraint, start–end position constraint, and start–end velocity constraint. By using the sequential convex optimization and first-order Taylor approximation, the nonconvex problem is transformed into a convex one. An iterative algorithm is proposed to solve the problem. Numerical results verify the efficiency of the proposed algorithm and also show the effects of buffer size on a UAV’s trajectory.

## 1. Introduction

Different from terrestrial static relay, unmanned aerial vehicle (UAV)-based relay has low cost and high mobility, which plays an important role in many application scenarios (such as emergency responses and delay-tolerant applications) [1]. The performance of UAV-assisted communication systems can be enhanced via dynamic UAV relocations. Therefore, the UAV-assisted wireless communications have attracted increasing interest.

Future wireless communications networks are required to meet a high rate requirement. However, the rapid growth of wireless traffic has led to radio frequency (RF) spectrum congestion. Free-space optical (FSO) communication is considered as an attractive solution due to its large bandwidth. Moreover, the narrow beams of FSO communications facilitate secure and interference-free communications. However, these advantages of FSO communication come at the expense of some challenges: (1) FSO communication relies on the availability of a line-of-sight (LoS), which introduces critical limitations for mobile nodes. (2) FSO systems have an unpredictable connectivity due to atmospheric turbulence and visibility limiting conditions. (3) The pointing of the transmitter toward the photodetector has to be adaptively adjusted to mitigate effects of building sway. To mitigate the unpredictable connectivity of FSO links, both the RF and FSO techniques can be utilized to constitute a hybrid system (i.e., so-called hybrid FSO/RF system). In such systems, both the advantages of RF and FSO links can be exploited. For terrestrial static relaying systems, end-to-end performance has been well analyzed [2,3,4]. However, only a few studies focus on hybrid FSO/RF systems with UAV mobile relays. For UAV enabled hybrid FSO/RF systems, ergodic capacity [5], outage probability [6], ergodic sum rate [7], and throughput [8] were investigated. However, in [5,6,7,8], the energy consumption was not considered, which is a major challenge that limits a UAV’s flight time. To prolong the operation time, the authors considered a solar-powered UAV [9]. In [10], the energy efficiency for a UAV-based RF system was analyzed.

Due to the size and weight constraints, the UAV’s on-board energy is finite, which will limit the UAV’s flight time. An improvement in energy efficiency directly increases the amount of information bits that can be communicated with the UAV before it needs to be recalled for recharging/refueling. Therefore, energy-efficient trajectory optimization for maximizing the information bits per unit energy consumption of the UAV is important. To make full use of the UAVs’ high maneuverability, researchers have concentrated on optimizing the trajectory of the UAVs. For a UAV-based dual-hop RF relaying system, the UAV’s trajectory was optimized by minimizing the energy consumption [11] or maximizing the UAV’s flight time [12]. In mixed FSO/RF systems, the rate imbalance problem caused by using different types of links is a major concern, the buffer-assisted UAV relaying was proposed [10,13]. Ref. [14] demonstrated that the buffer can enhance system performance. To the best of our knowledge, the energy-efficient trajectory optimization for ahybrid FSO/RF system with a buffer-aided UAV has not been studied.

In this paper, we will analyze the energy efficiency and then optimize the UAV’s trajectory for a hybrid FSO/RF communication system. The main contributions of the paper are listed as follows:

- Different from [9,10,11,12] focusing on the UAV based RF scenarios, we consider a UAV based hybrid FSO/RF system with a buffer, which is a promising solution to the emerging wireless backbone network. Unlike [5,6,7,8], we focus on analyzing the energy efficiency of the system. Initially, we obtain an energy consumption model, which includes communication-related energy consumption and propulsion energy consumption. Then, the throughput is derived as the total data rate of reaching the destination. Finally, the energy efficiency is derived as the throughput normalized by the energy consumption.
- Based on the derived energy efficiency expression, we optimize the UAV’s trajectory by maximizing energy efficiency under the buffer constraint, velocity constraint, acceleration constraint, start–end position constraint, and start–end velocity constraint. The considered optimization problem is nonconvex. By using the sequential convex optimization and first-order Taylor approximation, we transform the nonconvex problem into a convex problem and propose an iterative algorithm to solve the problem. To the best of our knowledge, there is no other literature to tackle the energy-efficient trajectory optimization of such a hybrid system.
- For different scenarios, simulation results for energy efficiency maximized trajectories are provided. It is shown that the proposed iterative algorithm can effectively alleviate the data rate imbalance of the two links and obtain good energy efficiency. Moreover, the proposed algorithm always outperforms the existing scheme. Therefore, the proposed algorithm can be utilized for the practical implementation of the UAV-based hybrid FSO/RF system.

The reminder of this paper is organized as follows. Section 2 introduces the system model. In Section 3, the energy efficiency is analyzed. In Section 4, the UAV’s trajectory is optimized. Some numerical results are provided in Section 5. Finally, Section 6 concludes the paper.

## 2. System Model

**Figure 1:**
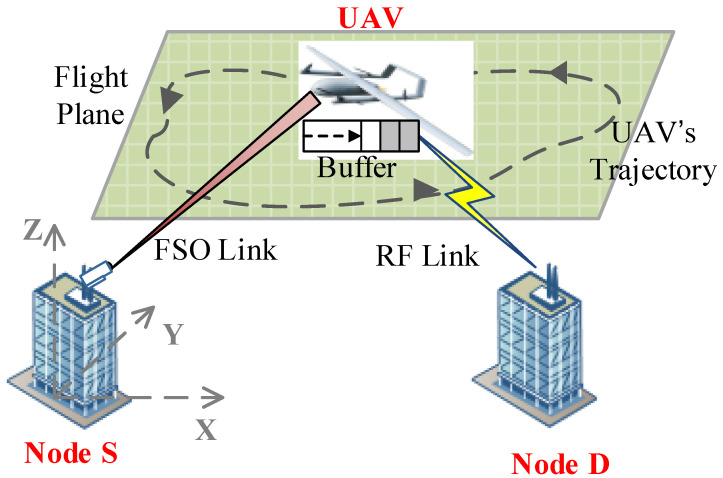
A hybrid FSO/RF system with a UAV-assisted mobile relay.

Consider a hybrid FSO/RF communication system with a fixed-wing UAV relay, as shown in Figure 1. In the system, the source (node S) and destination (node D), which are far away from each other, are fixed nodes. To improve the communication quality of the two nodes, a UAV-assisted relay node with decode–and–forward protocol is employed. By employing the UAV, the whole system is divided into two hops. The first hop is the FSO link, while the second hop is the RF link. At the UAV, it decodes the signal and puts it into a buffer. Note that the buffer is utilized to describe the queuing system of a practical UAV relay. Then, the UAV re-encodes the information and forwards it to node D by employing RF communication technology in the second hop.

We consider a three-dimensional Cartesian coordinate system, the coordinates of node S, node D, and the UAV are set to be

(1) $$\begin{array}{c} \left\{\begin{array}{c} q_{S}={\left(0,0,H_{S}\right)}^{T}\\ q_{D}={\left(L,0,H_{D}\right)}^{T}\\ q_{R}\left(t\right)={\left(x_{R}\left(t\right),y_{R}\left(t\right),H_{R}\right)}^{T} \end{array}\right., \end{array}$$

where $H_{S}$ and $H_{D}$ denote the altitudes of node S and node D; *L* denotes the horizontal distance between node S and node D; $H_{R}$ denotes the constant altitude of the UAV (To facilitate the analysis, the UAV’s altitude is set to be a constant, and the UAV’s trajectory is two-dimensional. Actually, we can extend the UAV trajectory design to be a three-dimensional one by setting the UAV’s altitude to be a variable); and $x_{R}\left(t\right)$ and $y_{R}\left(t\right)$ denote *X*-axis and *Y*-axis coordinates of the UAV, which vary with time t(t∈[0,T]).

The UAV’s trajectory is well characterized by its location $q_{R}\left(t\right)$, velocity $v_{R}\left(t\right)$, and acceleration $a_{R}\left(t\right)$ with t∈[0,T]. To facilitate the analysis, the period *T* is discretized into *N* slots with step size $\delta_{t}$, i.e., $t=n\delta_{t},\;\forall n\in \left\{0,1,\ldots ,N\right\}$. Consequently, the UAV’s trajectory is recharacterized by

(2) $$\begin{array}{c} \left\{\begin{array}{c} q_{R}\left[n\right]=q_{R}\left(n\delta_{t}\right)\\ v_{R}\left[n\right]=v_{R}\left(n\delta_{t}\right)\\ a_{R}\left[n\right]=a_{R}\left(n\delta_{t}\right) \end{array}\right.,\;\forall n. \end{array}$$

For any infinitesimal step size $\delta_{t}$, by the first order and second order Taylor approximations, we obtain a discrete state–space model as [10]

(3) $$\begin{array}{c} \left\{\begin{array}{c} v_{R}\left[n+1\right]=v_{R}\left[n\right]+a_{R}\left[n\right]\delta_{t}\\ q_{R}\left[n+1\right]=q_{R}\left[n\right]+v_{R}\left[n\right]\delta_{t}+\frac{1}{2}a_{R}\left[n\right]\delta_{t}^{2} \end{array}\right.,\;\forall n. \end{array}$$

For the FSO link, we assume that node S is equipped with an optical tracking aligner to counteract the effects of UAV’s motion and beam jitter, and the line-of-sight (LoS) link always exists. Therefore, the signal attenuation mainly depends on atmospheric attenuation, i.e., [15]

(4) $$\begin{array}{c} h_{\mathrm{FSO}}\left[n\right]=\exp\left(-\Phi Z_{\mathrm{SR}}\left[n\right]\right),\forall n, \end{array}$$

where Φ is the attenuation coefficient, and $Z_{\mathrm{SR}}\left[n\right]=\sqrt{x_{R}^{2}\left[n\right]+y_{R}^{2}\left[n\right]+{\left(H_{R}-H_{S}\right)}^{2}}$ is the distance between node S and the UAV.

For the RF link, we assume that the LoS from the UAV to node D always exists. This is reasonable because the air link is more likely to have a LoS link compared to the ground link. Moreover, the fast fading that may occur with the location and movement of the UAV is perfectly compensated [12]. Therefore, the RF channel is mainly characterized by path loss, which can be expressed as

(5) $$\begin{array}{c} h_{\mathrm{RF}}\left[n\right]=\beta_{0}Z_{\mathrm{RD}}^{-\eta }\left[n\right],\forall n, \end{array}$$

where $\beta_{0}$ is the reference channel gain at $d_{0}=1\;m$, η is path loss exponent, and $Z_{\mathrm{RD}}\left[n\right]=\sqrt{\left(L-x_{R}^{2}\left[n\right]\right)+y_{R}^{2}\left[n\right]+{\left(H_{R}-H_{D}\right)}^{2}}$ is the distance of the RF link.

Note that large-scale fading is necessary for effective network deployment, while small-scale fading is important for the physical-layer designs to develop and test different transmission strategies [16]. In this paper, we focus on the optimization of UAV’s trajectory, which falls within the scope of network deployment, and thus small-scale fading is not considered in (4) and (5).

## 3. Performance Analysis

In this section, the energy consumption and the throughput will be modeled. Moreover, the energy efficiency of the system will be analyzed.

### 3.1. Energy Consumption

The total energy consumption of the UAV includes two components: communication-related energy consumption and propulsion energy consumption.

The communication-related energy consumption is caused by the radiation, signal processing, and other circuitry, which is given by

(6) $$E_{c}\left[n\right]=P_{c}\delta_{t},\forall n,$$

where $P_{c}$ denotes the communication power of the UAV.

The propulsion energy should also be considered to guarantee that the UAV remains aloft and keeps moving. For the fixed-wing UAV with level flight under normal operations, the propulsion energy consumption is a function of the trajectory, which is expressed as [10]

(7) $$\begin{array}{ccc} E_{p}\left[n\right] & = & \left[c_{1}{∥v_{R}\left[n\right]∥}^{3}+\frac{c_{2}}{∥v_{R}\left[n\right]∥}\left(1+\frac{{∥a_{R}\left[n\right]∥}^{2}-\frac{{\left(a_{R}^{T}\left[n\right]v_{R}\left[n\right]\right)}^{2}}{{∥v_{R}\left[n\right]∥}^{2}}}{g^{2}}\right)\right]\;\;\delta_{t}\\ & + & \frac{1}{2}m\left({∥v_{R}\left[n\right]∥}^{2}-{∥v_{R}\left[n-1\right]∥}^{2}\right),\forall n, \end{array}$$

where $c_{1}$ and $c_{2}$ are two parameters related to the UAV’s weight, wing area, and air density; *g* is the gravitational acceleration; and *m* is mass of the UAV.

According to (6) and (7), the total energy consumption over the period *T* can be expressed as

(8) $$\begin{array}{ccc} E_{T}\left(q_{R}\left[n\right],v_{R}\left[n\right],a_{R}\left[n\right]\right) & = & \sum_{n=1}^{N}\left[c_{1}{∥v_{R}\left[n\right]∥}^{3}+\frac{c_{2}\left(g^{2}+{∥a_{R}\left[n\right]∥}^{2}-\frac{{\left(a_{R}^{T}\left[n\right]v_{R}\left[n\right]\right)}^{2}}{{∥v_{R}\left[n\right]∥}^{2}}\right)}{∥v_{R}\left[n\right]∥g^{2}}\right]\delta_{t}\\ & + & \underset{\overset{\Delta }{\;=}\Delta_{K}}{\underset{︸}{\frac{m}{2}\left({∥v_{R}\left[N\right]∥}^{2}-{∥v_{R}\left[0\right]∥}^{2}\right)}}+P_{c}N\delta_{t}\;\left[\mathrm{Joule}\right], \end{array}$$

where $\Delta_{K}$ is the kinetic energy difference, and it is fixed when the initial and final locations of the UAV are preselected.

To facilitate the trajectory optimization, the total energy consumption (8) is upper bounded by

(9) $$\begin{array}{ccc} E_{T}\left(q_{R}\left[n\right],v_{R}\left[n\right],a_{R}\left[n\right]\right) & \leq & \sum_{n=1}^{N}\left[c_{1}{∥v_{R}\left[n\right]∥}^{3}+\frac{c_{2}}{∥v_{R}\left[n\right]∥}\left(1+\frac{{∥a_{R}\left[n\right]∥}^{2}}{g^{2}}\right)\right]\delta_{t}\\ & + & \underset{≜\Delta_{K}}{\underset{︸}{\frac{m}{2}\;\left({∥v_{R}\left[N\right]∥}^{2}-{∥v_{R}\left[0\right]∥}^{2}\right)}}+P_{c}N\delta_{t}\;\left[\mathrm{Joule}\right]. \end{array}$$

### 3.2. Throughput

The achievable rate of the FSO link is given by [17]

(10) $$R_{\mathrm{FSO}}\left[n\right]=\frac{B_{\mathrm{FSO}}}{2\log_{2}e}\log_{2}\left(1+\zeta e^{-2\Phi ∥q_{R}\left[n\right]-q_{S}∥}\right),\forall n,$$

where $B_{\mathrm{FSO}}$ is the bandwidth of the FSO link, and ζ is given by [17]

(11) $$\begin{array}{c} \zeta =\left\{\begin{array}{c} \frac{e^{2\alpha \mu^{*}}\gamma_{\mathrm{FSO}}^{2}}{2\pi e\alpha^{2}}{\left(\frac{1-e^{-\mu^{*}}}{\mu^{*}}\right)}^{2},\;\;\;0<\alpha <\frac{1}{2}\\ \frac{\gamma_{\mathrm{FSO}}^{2}}{2\pi e\alpha^{2}},\;\;\;\;\;\;\;\;\;\;\;\;\;\;\;\;\frac{1}{2}\leq \alpha <1 \end{array}\right., \end{array}$$

and $\gamma_{\mathrm{FSO}}^{2}=P_{\mathrm{FSO}}^{2}/\sigma_{\mathrm{FSO}}^{2}$, $\alpha =P_{\mathrm{FSO}}/\Lambda$, where $\sigma_{\mathrm{FSO}}^{2}$ is the noise power, and $P_{\mathrm{FSO}}$ and Λ are the average power and peak power of node S. $\mu^{*}$ is the solution to $\frac{1}{\mu^{*}}-\frac{e^{-\mu^{*}}}{1-e^{-\mu^{*}}}=\alpha$.

We assume that a constant RF transmission power $P_{\mathrm{RF}}$ is employed by the UAV, which corresponds to the maximum allowable value imposed by the authority regulations. Accordingly, the achievable rate of the RF link is expressed as [18]

(12) $$R_{\mathrm{RF}}\left[n\right]=B_{\mathrm{RF}}\log_{2}\left(1+\frac{\gamma_{\mathrm{RF}}}{Z_{\mathrm{RD}}^{\eta }\left[n\right]}\right),\forall n,$$

where $\gamma_{\mathrm{RF}}\;=\;P_{\mathrm{RF}}\beta_{0}/\sigma_{\mathrm{RF}}^{2}$ indicates the reference signal-to-noise ratio at distance d=1m, $B_{\mathrm{RF}}$ denotes the RF bandwidth, and $\sigma_{\mathrm{RF}}^{2}$ denotes the noise power.

For the FSO link, the practical rate $R_{\mathrm{FSO}}^{\mathrm{prac}}[n]$ satisfies $R_{\mathrm{FSO}}^{\mathrm{prac}}[n]\leq R_{\mathrm{FSO}}\left[n\right],\forall n$. Note that the buffer will overflow when the written data overrun the buffer’s boundary. Therefore, the overflowed data are invalid, which should be retransmitted in the next time slot. Only the non-overflowed data are received by the UAV.

We assume that node S transmits data to the UAV with rate $R_{\mathrm{FSO}}^{\mathrm{prac}}[n]$, and the UAV receives the data and stacks them into the buffer by the first-in-first-out scheme. Thus, the remaining bits in the buffer are given by

(13) $$Q\left[n\right]=Q\left[n-1\right]+R_{\mathrm{FSO}}^{\mathrm{prac}}[n]\delta_{t}-R_{\mathrm{RF}}^{\mathrm{prac}}[n]\delta_{t},\;1\leq n\leq N,$$

where Q[0]=0 denotes the initial state of the buffer, and $R_{\mathrm{RF}}^{\mathrm{prac}}\left[n\right]$ is the practical rate of RF link. Without loss of generality, the full-duplex communication is employed, i.e., the RF link also works when the UAV receives data from node S (For a half-duplex relay, it needs two orthogonal time slots to transfer the data from source node to destination node, resulting in loss of the spectral efficiency. In this paper, the full-duplex UAV relay can simultaneously receive from source node and transmit to destination node. As a result, the data bits in the buffer can be processed more efficiently, and the throughput of the system can also be improved). Therefore, $R_{\mathrm{RF}}^{\mathrm{prac}}\left[n\right]$ can be written as

(14) $$R_{\mathrm{RF}}^{\mathrm{prac}}\left[n\right]=\min\left\{\frac{Q[n-1]}{\delta_{t}},R_{\mathrm{RF}}\left[n\right]\right\},\;\;\;2\leq n\leq N,$$

with $R_{\mathrm{RF}}^{\mathrm{prac}}\left[1\right]=0$.

The total throughput is the rate of arriving at node D, i.e.,

(15) $$\tau \left(q_{R}\left[n\right]\right)=\sum_{i=1}^{N}R_{\mathrm{RF}}^{\mathrm{prac}}\left[i\right]\delta_{t}\;\left[\mathrm{bits}\right].$$

### 3.3. Energy Efficiency

According to (9) and (15), we can obtain the energy efficiency as

(16) $$EE\left(q_{R}\left[n\right],v_{R}\left[n\right],a_{R}\left[n\right]\right)=\frac{\tau (q_{R}\left[n\right])}{E_{T}\left(q_{R}\left[n\right],v_{R}\left[n\right],a_{R}\left[n\right]\right)}\;\;\;[\mathrm{bits}/\mathrm{Joule}].$$

## 4. Trajectory Optimization of UAV

### 4.1. Problem Formulation

Before formulating the optimization problem, we will introduce the constraints that should be considered in the system.

Generally, the FSO link provides a higher data rate than the RF link. Considering such a data rate imbalance, we employ a buffer at the UAV to avoid data overflow. Moreover, the remaining bits in the buffer at each time slot should be nonnegative. Thus, the buffer constraint is given by

(17) $$0\leq Q\left[n\right]\leq Q_{\max},\;1\leq n\leq N,$$

where $Q_{\max}$ is the buffer size.

The initial position (i.e., start position) $q_{I}$ and final position (i.e., end position) $q_{F}$ can be UAV bases or energy supply locations, which are determined in advance. Thus, the initial and final values of $q_{R}\left[n\right]$ are set as

(18) $$q_{R}\left[0\right]=q_{I},\;q_{R}\left[N\right]=q_{F}.$$

Similarly, the initial velocity (i.e., start velocity) $v_{I}$ and final velocity (i.e., end velocity) $v_{F}$ are also given in advance, and $v_{R}\left[0\right]$ and $v_{R}\left[N\right]$ are set as

(19) $$v_{R}\left[0\right]=v_{I},\;v_{R}\left[N\right]=v_{F}.$$

Considering the flow control and performance limitation, the UAV’s velocity is limited. Therefore, the velocity constraint is modeled as

(20) $$v_{\min}\leq ∥v_{R}\left[n\right]∥\leq v_{\max},\;\forall n,$$

where $v_{\max}$ and $v_{\min}$ are the maximum and minimum velocities. Similarly, the acceleration constraint is given by

(21) $$∥a_{R}\left[n\right]∥\leq a_{\max},\;\forall n,$$

where $a_{\max}$ is the maximum acceleration of UAV.

The objective of this paper is to find the optimal trajectory of the UAV by maximizing the energy efficiency under constraints (3), (17)–(21). Mathematically, the energy-efficient optimization problem is formulated as

(22) $$\begin{array}{ccc} & \max_{q_{R}\left[n\right],v_{R}\left[n\right],a_{R}\left[n\right]}\;\; & EE(q_{R}\left[n\right],v_{R}\left[n\right],a_{R}\left[n\right]),\\ & s.t. & \;(3),\;(17)–(21). \end{array}$$

Despite some convex constraints, the nonconcave objective function and nonconvex constraints (17) and (20) make the problem a nonconvex one. It is challenging to solve such a nonconvex problem with standard convex optimization methods.

### 4.2. Problem Solving

Because problem (22) is neither a convex nor quasiconvex problem, Slater’s condition is not employed here. Alternatively, we employ the sequential convex optimization. According to Proposition 3 in [19], it is known that the sequences ${\left\{q_{R}\left[n\right],\;v_{R}\left[n\right],\;a_{R}\left[n\right]\right\}}_{n=1}^{N}$ converge to a point fulfilling the Karush-Kuhn-Tucker (KKT) optimality conditions of the primal nonconvex problem (22). This implies that at least a local optimal solution can be found for the problem (22). Due to the nonconvexity of problem (22), we do not focus on the KKT conditions, but employ the sequence convex optimization, local convex approximation, and fractional optimization in this subsection to solve the problem.

By introducing a group of slack variables λ[n],∀n, problem (22) is reformulated as

(23) maxqR[n],vR[n],aR[n],λ[n]∑n=2NBRFlog21+γRF∥qR[n]−qD∥2∑n=1Nc1∥vR[n]∥3+c2λ[n]1+∥aR[n]∥2g2+ΔKδt+PcNs.t.(3),(17)–(19),(21),C1:∥vR[n]∥≤vmax,∀nC2:λ[n]≥vmin,∀nC3:∥vR[n]∥2≥λ[n]2,∀n.

**Remark** **1.** *Problem (23) is equivalent to problem (22). This is because we must obtain $\lambda \left[n\right]=∥v_{R}\left[n\right]∥,\forall n$ at the optimal solution of problem (23); otherwise, we can increase λ[n] to obtain a larger objective value.*

Now, the denominator of the objective function in problem (23) is jointly convex with respect to $\left\{v_{R}\left[n\right],a_{R}\left[n\right],\lambda \left[n\right]\right\}$ but with a new nonconvex constraint $C_{3}$. To tackle constraint $C_{3}$, we employ a local convex approximation. Note that $∥v_{R}\left[n\right]{∥}^{2}$ is a differentiable and convex function of $v_{R}\left[n\right]$. For any local point $\left\{v_{R}^{j}\left[n\right]\right\}$ obtained at the jth iteration, we obtain

(24) $$\begin{array}{c} ∥v_{R}\left[n\right]{∥}^{2}\geq \underset{\overset{\Delta }{\;=}\phi_{\mathrm{lb}}\left(∥v_{R}^{j}\left[n\right]∥\right)}{\underset{︸}{{∥v_{R}^{j}\left[n\right]∥}^{2}+2{\left(v_{R}^{j}\left[n\right]\right)}^{T}\left(v_{R}\left[n\right]-v_{R}^{j}\left[n\right]\right)}}. \end{array}$$

The equality holds when $v_{R}\left[n\right]=v_{R}^{j}\left[n\right]$. Moreover, at the local point $v_{R}^{j}\left[n\right]$, both $∥v_{R}\left[n\right]{∥}^{2}$ and $\phi_{\mathrm{lb}}\left(∥v_{R}^{j}\left[n\right]∥\right)$ have the same gradient (i.e., $2v_{R}^{j}\left[n\right]$). Then, define a new constraint

(25) $$\phi_{\mathrm{lb}}\left(∥v_{R}^{j}\left[n\right]∥\right)\geq \lambda {\left[n\right]}^{2},\;\forall n,$$

which is convex because $\phi_{\mathrm{lb}}\left(∥v_{R}^{j}\left[n\right]∥\right)$ is linear with $v_{R}\left[n\right]$. From (24) and (25), it is known that the convex constraint (25) always implies the nonconvex constraint $C_{3}$.

Then, to tackle the nonconcavity of the numerator of the objective function in problem (23) and nonconvexity of constraint (17), we introduce the first-order Taylor approximation for a local point at the jth iteration. Define a lower bound of throughput for the RF link as

(26) $$\begin{array}{ccc} R_{\mathrm{RF}}^{j}\left[n\right] & = & B_{\mathrm{RF}}\left[\log_{2}\left(1+\frac{\gamma_{\mathrm{RF}}}{{∥q_{R}^{j}\left[n\right]-q_{D}∥}^{2}}\right)\right.\\ & - & \left.\frac{\left(\log_{2}e\right)\gamma_{\mathrm{RF}}\left({∥q_{R}\left[n\right]-q_{D}∥}^{2}-{∥q_{R}^{j}\left[n\right]-q_{D}∥}^{2}\right)}{\left({∥q_{R}^{j}\left[n\right]-q_{D}∥}^{2}+\gamma_{\mathrm{RF}}\right)\left({∥q_{R}^{j}\left[n\right]-q_{D}∥}^{2}\right)}\right], \end{array}$$

where $q_{R}^{j}\left[n\right]$ is a local point obtained at the jth iteration. Similarly, define a lower bound of throughput for the FSO link as

(27) $$\begin{array}{ccc} R_{\mathrm{FSO}}^{j}\left[n\right] & = & \frac{B_{\mathrm{FSO}}}{2\log_{2}e}\left[\log_{2}\left(1+\zeta e^{-2\Phi ∥q_{R}^{j}\left[n\right]-q_{S}∥}\right)\right.\\ & - & \frac{2\Phi \zeta }{e^{2\Phi ∥q_{R}^{j}\left[n\right]-q_{S}∥}+\zeta }\left.\left({∥q_{R}\left[n\right]-q_{S}∥}^{2}-{∥q_{R}^{j}\left[n\right]-q_{S}∥}^{2}\right)\right]. \end{array}$$

In particular, by high signal-to-noise ratio approximation, the lower-bounded throughput of the FSO link is further written as

(28) $$R_{\mathrm{FSO}}^{j}\left[n\right]=\frac{B_{\mathrm{FSO}}}{2\log_{2}e}\left[\log_{2}\zeta -2\Phi \left(∥q_{R}\left[n\right]-q_{S}∥\right)\right].$$

It is noted that $R_{\mathrm{FSO}}^{j}\left[n\right]$ and $R_{\mathrm{RF}}^{j}\left[n\right]$ are concave functions with $q_{R}\left[n\right]$. Then, we introduce slack variables $\ell_{1}\left[n\right]$, $\ell_{2}\left[n\right]$ and replace constraint (17) with a convex constraint. Therefore, we can recursively rewrite the queue state as

(29) $$Q^{\prime }\left[n\right]=\sum_{i=1}^{n}\ell_{1}\left[n\right]\delta_{t}-\sum_{i=2}^{n}\ell_{2}\left[n\right]\delta_{t},n=2,\cdots ,N.$$

For any given local value $q_{R}^{j}\left[n\right]$ at the jth iteration, we can reformulate problem (23) as

(30) maxqRj[n],vRj[n],aRj[n],λ[n],ℓ1[n],ℓ2[n]∑n=2Nℓ2[n]∑n=1Nc1∥vRj[n]∥3+c2λ[n]1+∥aRj[n]∥2g2+ΔKδt+PcNs.t.(3),(19),(21),(25),C1,C2,C4:0≤ℓ1[n]≤RFSOj[n],n=1,⋯,NC5:0≤ℓ2[n]≤RRFj[n],n=2,⋯,NC6:0≤Q′[n]≤Qmax,n=1,⋯,N.

Problem (30) is a fractional maximization problem with a concave numerator, a convex denominator, and all convex constraints, which can be solved by classic bisection method [20] or Dinkelbach’s algorithm [21]. The convergence rate of bisection method is linear, while the convergence rate of Dinkelbach’s algorithm is superlinear. Therefore, Dinkelbach’s algorithm is employed to solve problem (30).

To solve the original problem (22), we propose Algorithm 1 to iteratively optimize problem (30) with the local point ${\left\{q_{R}^{j}\left[n\right],\;v_{R}^{j}\left[n\right],\;a_{R}^{j}\left[n\right]\right\}}_{n=1}^{N}$ updated in each iteration. Let the complexity of Dinkelbach’s algorithm be O(Ξ) and the maximum iteration number be $I_{\max}$, the total complexity of Algorithm 1 is $O(\Xi I_{\max})$, which indicates that the proposed algorithm is time-efficient.
**Algorithm 1** Proposed iterative algorithm.1:**Input:** Basic simulation parameters;2:**Output:**${\left\{q_{R}^{*}\left[n\right],\;v_{R}^{*}\left[n\right],\;a_{R}^{*}\left[n\right]\right\}}_{n=1}^{N}$;3:**Initialize:**${\left\{q_{R}^{0}\left[n\right],\;v_{R}^{0}\left[n\right],\;a_{R}^{0}\left[n\right]\right\}}_{n=1}^{N}$, and set j=0;4:**While** ($j\leq I_{\max}$) **do**5:   Solve problem (30) by using Dinkelbach’s algorithm for the local points ${\left\{q_{R}^{j}\left[n\right],\;v_{R}^{j}\left[n\right],\;a_{R}^{j}\left[n\right]\right\}}_{n=1}^{N}$, and find the optimal solution ${\left\{q_{R}^{*}\left[n\right],\;v_{R}^{*}\left[n\right],\;a_{R}^{*}\left[n\right]\right\}}_{n=1}^{N}$;6:   Update j=j+1;7:   Update $q_{R}^{j}\left[n\right]=q_{R}^{*}\left[n\right],\;v_{R}^{j}\left[n\right]=v_{R}^{*}\left[n\right],\;a_{R}^{j}\left[n\right]=a_{R}^{*}\left[n\right]$;8:**EndWhile**

## 5. Numerical Results

In this section, numerical results are provided to validate the proposed algorithm. The basic simulation parameters are listed in Table 1. Furthermore, we initialize the UAV’s trajectory as a uniform linear motion with $v_{I}=v_{F}=\left(q_{F}-q_{I}\right)/T$.

**Figure 2:**
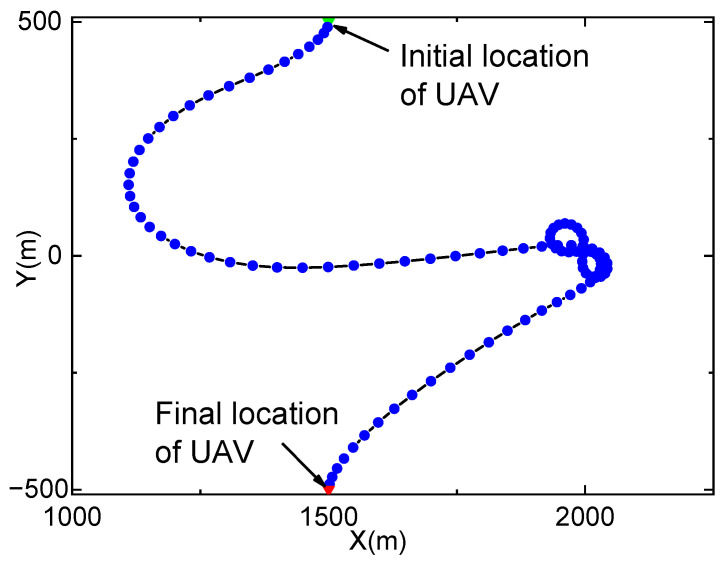
Optimized trajectory of the UAV when $Q_{\max}=\infty$.

**Figure 3:**
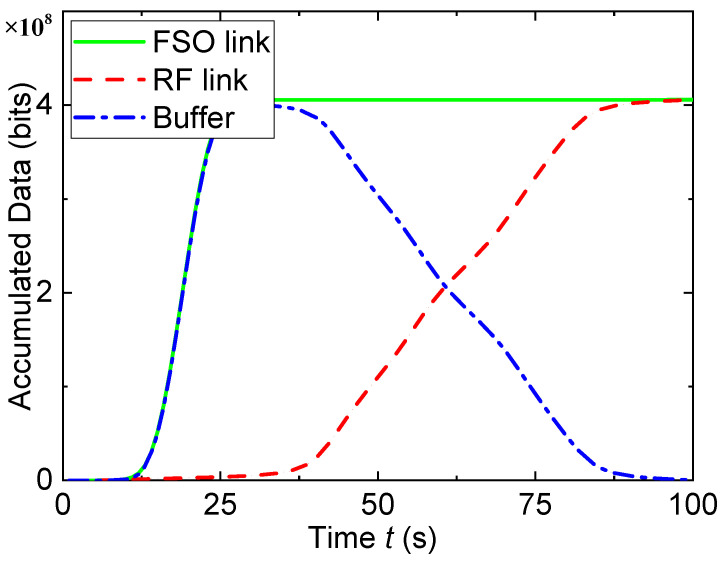
Accumulated data when $Q_{\max}=\infty$.

Figure 2 and Figure 3 show the optimized trajectory of the UAV and the accumulated data when $Q_{\max}=\infty$. In Figure 2 the UAV first flies from the initial location to the area closing to node S to fill data into the buffer, and then moves to the area closing to node D to forward data accumulated in the buffer. It then hovers over Node D in the shape of the number “8” and finally files to the final location. Because the buffer size is infinite, the buffer will never overflow, and the UAV does not move back and forth between node S and node D. In Figure 3, when *t* is small, both the accumulated data bits of the FSO link and the buffer increase rapidly with the increase in *t*. In this period, the throughput of the RF link is low, and thus the accumulated data bits of the RF link are small. As the UAV moves to node D, the accumulated data of the RF link continue to increase after the accumulated data in the buffer reach $4\times 10^{8}$ bits, which corresponds to the “8”-shaped trajectory of the UAV in Figure 2. It is noted that the accumulated data in the buffer are almost exhausted after the UAV completes its relay task (i.e., when t=T, which validates the efficiency of the proposed optimization algorithm.

**Figure 4:**
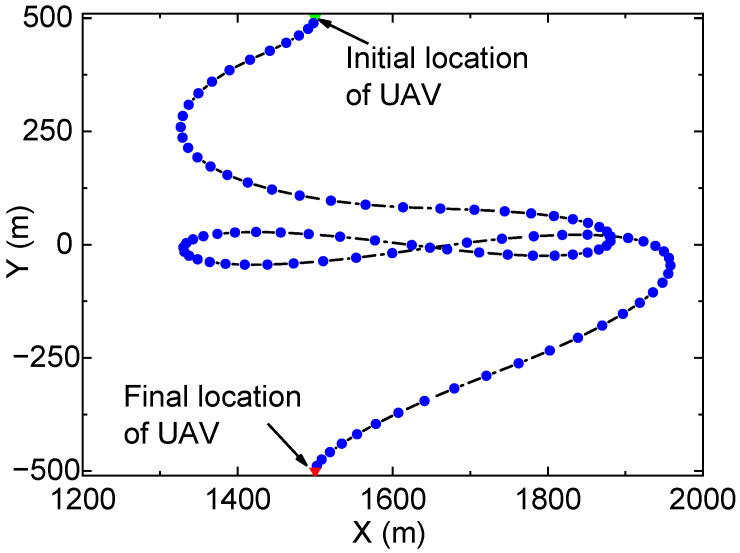
Optimized trajectory of the UAV when $Q_{\max}=10^{7}\;\mathrm{bits}$.

**Figure 5:**
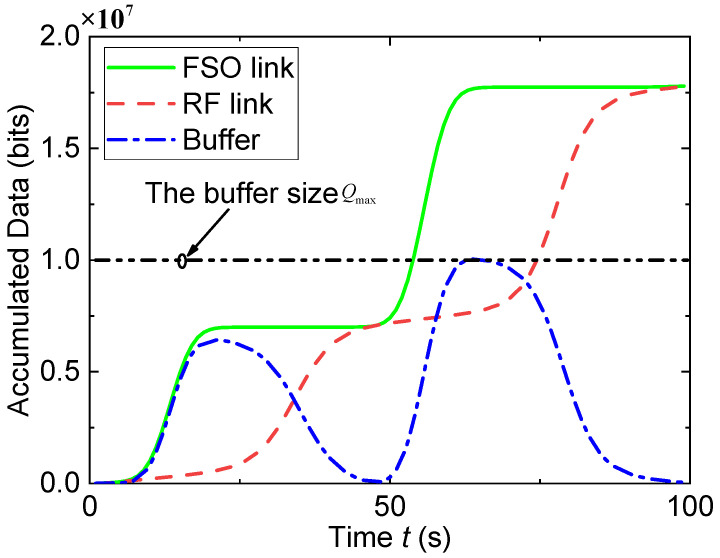
Accumulated data when $Q_{\max}=10^{7}\;\mathrm{bits}$.

Figure 4 and Figure 5 show the UAV’s trajectory and the accumulated data for a finite buffer size (i.e., $Q_{\max}=10^{7}\;\mathrm{bits}$). Unlike Figure 2, the UAV in Figure 4 flies back and forth quickly between node S and node D to avoid buffer overflow. In Figure 5, the accumulated data of the FSO link show two fast increasing trends, which corresponds to the two periods when the UAV approaches node S in Figure 4. Similarly, the two rapid increasing trends of accumulated data in the RF link correspond to the two periods when the UAV approaches node D in Figure 4. Moreover, the data in the buffer do not exceed the buffer size, which validates the constraint $Q^{\prime }\left[n\right]\leq Q_{\max}$. Similar to Figure 3, the buffered data are almost exhausted finally, which verifies the efficiency of the proposed algorithm.

**Figure 6:**
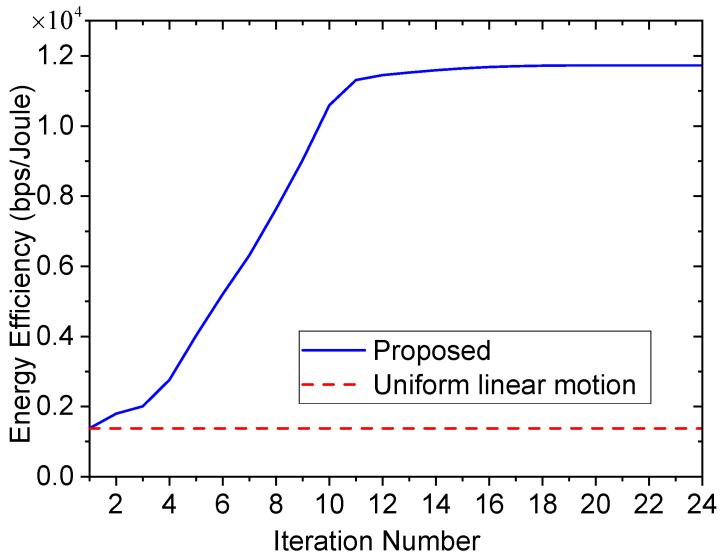
Energy efficiency of different algorithms.

Figure 6 shows the energy efficiency of different algorithms versus iteration number. To facilitate the comparison, the uniform linear motion algorithm is employed as the benchmark. In the uniform linear motion algorithm, the UAV flies with a constant velocity, and the UAV’s trajectory is a straight line connecting the initial and final locations. As can be seen, the proposed algorithm always outperforms the uniform linear motion algorithm, which verifies the efficiency of the proposed algorithm.

## 6. Conclusions

For a UAV-assisted hybrid FSO/RF system, this paper studied the optimal trajectory of the UAV via the energy efficiency maximization. Initially, the energy efficiency of the system was analyzed. Then, a nonconvex optimization problem was proposed, which was transformed to a convex one by using the sequential convex optimization and first-order Taylor approximation. Finally, an iterative algorithm was proposed to solve the problem. Through the simulation results, the proposed algorithm can effectively alleviate the rate imbalance of the two links and obtain a good energy efficiency, and thus can be utilized for practical system implementation.

## Figures and Tables

**Table 1:** Basic simulation parameters.

Parameters	Symbols	Values
Weight of UAV	*m*	100kg
Gravity acceleration	*g*	$9.8\;m^{2}/s$
Location of node S	$q_{S}$	${\left[0\;m,0\;m,0\;m\right]}^{T}$
Location of node D	$q_{D}$	${\left[2000\;m,0\;m,0\;m\right]}^{T}$
Initial location of UAV	$q_{I}$	$[1500\;m,500\;m,100\;{m]}^{T}$
Final location of UAV	$q_{F}$	$[1500\;m,-500\;m,100\;{m]}^{T}$
Maximum velocity of UAV	$v_{\max}$	100m/s
Minimum velocity of UAV	$v_{\min}$	3m/s
Maximum acceleration of UAV	$a_{\max}$	$5\;m/s^{2}$
UAV’s parameters	$c_{1},c_{2}$	$9.26\times 10^{-4}\;\mathrm{kg}/m$, $2250\;\mathrm{kg}\;m^{3}/s^{4}$
Total communication consumption of UAV	$P_{c}$	10W
FSO link parameters	$B_{\mathrm{FSO}}$, α, $P_{\mathrm{FSO}}$, $\sigma_{\mathrm{FSO}}^{2}$, Φ	$10^{8}\;\mathrm{Hz}$, 0.5, 0.2W, $10^{-13}\;W$, 4.3dB/km
RF link parameters	$B_{\mathrm{RF}}$, $P_{\mathrm{RF}}$, $\sigma_{\mathrm{RF}}^{2}$, $\beta_{0}$, η	$10^{6}\;\mathrm{Hz}$, 0.01W, $10^{-11}\;W$, −5dB, 2
Time period	*T*	100s
Time-step size	$\delta_{t}$	1s

## Data Availability

Not applicable.
